# Revision of the Structure of Acremine P from a Marine-Derived Strain of *Acremonium persicinum*

**DOI:** 10.3390/molecules22040521

**Published:** 2017-03-24

**Authors:** Mary J. Garson, Warren Hehre, Gregory K. Pierens, Suciati

**Affiliations:** 1 School of Chemistry and Molecular Biosciences, University of Queensland, Brisbane, QLD 4072, Australia; 2 Wavefunction Inc., Irvine, CA 92612, USA; hehre@wavefun.com; 3 Centre for Advanced Imaging, University of Queensland, Brisbane, QLD 4072, Australia; greg.pierens@cai.uq.edu.au; 4 Faculty of Pharmacy, Airlangga University, Surabaya, East Java 60286, Indonesia; suciati@ff.unair.ac.id

**Keywords:** *Acremonium*, fungi, NOESY, DFT calculations, biosynthesis

## Abstract

The previously published structure of the fungal metabolite acremine P is revised by re-evaluation of chemical shift values and NOESY data, and by DFT calculations.

## 1. Introduction

**Chart 1:**
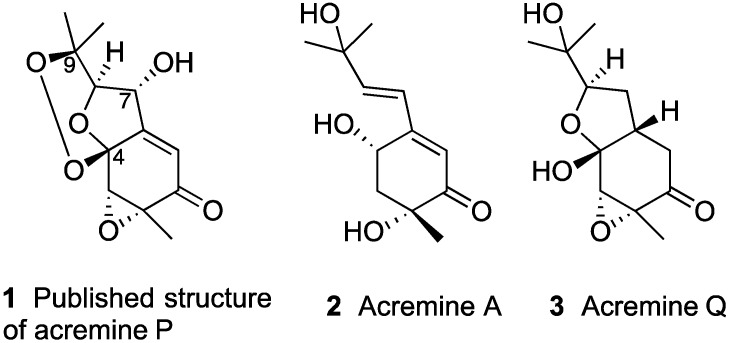
Selected meroterpenoids from *A. persicinum*.

Fungi from the genus *Acremonium* isolated from both terrestrial or marine sources have been reported to produce meroterpenoids, alkaloids, peptides or oxygenated metabolites [1,2,3,4,5,6]. Recently, we reported the structure elucidation, including a stereochemical investigation, of the meroterpenoid acremine P (**1**) (Chart 1) from a strain of *Acremonium persicinum* isolated from the marine sponge *Anomoianthella rubra*, obtained offshore from Mooloolaba in southeast Queensland. The chemical correlation of acremine P with its co-metabolite acremine A (**2**) by catalytic hydrogenation under mild conditions (H_2_, Pd/C, 24 h) was instrumental in defining the carbon framework and partial relative configuration of acremine P, while the absolute configuration at C-6 was determined by NMR analysis of *O-*methylmandelate (MPA) ester derivatives. The remaining stereochemical elements were resolved by evaluation of NOESY data, molecular modeling and selected heteronuclear coupling constant values [7].

In this communication, we revise the carbon framework of acremine P based on a comparison of calculated and experimental NMR chemical shift data. The predictions of chemical shift values by quantum chemical methods have provided valuable insights into natural product structures, including guiding the choice of diastereomer for structure confirmation by total synthesis [8,9,10]. Recent examples which highlight the role of computed chemical shift values in the stereochemical evaluation of natural products include vannusal B [11] and nobilisitine [12]. The structural revision of acremine A likewise necessitated a review of its overall relative stereochemistry, and this was informed by nOe data as well as molecular modeling. Finally, a biosynthetic pathway based on oxidative cleavage of a didehydro analogue of acremine Q is proposed.

## 2. Results and Discussions

The structures of acremines P and Q were examined using a combination quantum chemical/empirical approach (Spartan, Wavefunction Inc., Irvine, CA, USA) [13] that provides ^13^C-NMR shifts with a mean absolute error (MAE) of ~1.6 ppm. The calculations involve Boltzmann averaging from a ωB97X-V/6-311+G(2df,2p) model using conformer geometries from the ωB97X-D/6-31G* model and chemical shifts from ωB97X-D/6-31G*, but are also empirically corrected to account for the local environment. There was close agreement between the calculated vs. experimental ^13^C-NMR shift values for acremine Q (**3**), thereby corroborating the structure and stereochemistry of the metabolite as previously published. For acremine P, originally assigned structure **2**, the preliminary computational search for conformers revealed a single dominant conformer, with the closest alternative being upwards of 10 kJ/mol higher in energy and thus not contributing significantly to the equilibrium mixture. As shown in Table 1, there was a significant mismatching of the calculated vs. experimental ^13^C-NMR shift values for acremine P, with deviations of 20.4 ppm for the alkene carbon (C-2) and 23.0 ppm for the hydroxymethine carbon (C-7). Consequently, the published structure for acremine P could not be correct. While deviations of 3 ppm between the experimental and calculated values are considered acceptable, even up to 5–6 ppm for carbonyl groups, a deviation of >10 ppm between experimental and calculated values is diagnostic of an incorrect structure [8,14].

**Chart 2:**
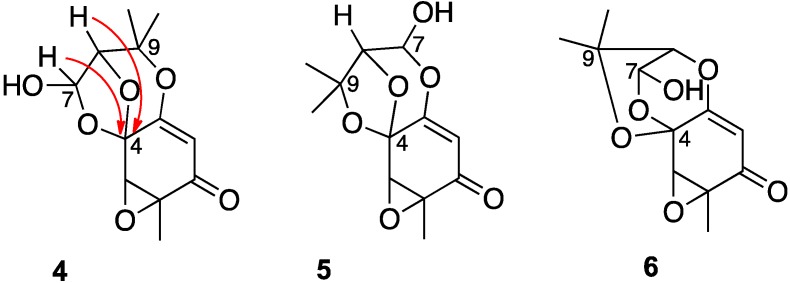
Candidate structures for acremine P.

A re-evaluation of the ^13^C-NMR shift values suggested that the signal at 95.0 ppm (C-7), previously assigned to a secondary alcohol center, was instead associated with an acetal or lactol center. Furthermore, the alkene carbon signals (102.4 and 162.5 ppm) indicated a polarized double bond, likely enolized given the number of oxygen atoms in the molecule. With this information, three candidate structures (**4**)–(**6**) (Chart 2), each of which contained a lactol functionality, were assessed against the previously reported HMBC data. In acremine P, the lactol proton at δ_H_ 5.83 (d) and the signal at ™_H_ 4.15 (s) for the hydroxymethine proton (H-8) each showed HMBC bond correlations to the acetal carbon at 99.0 ppm. In isomer **4**, these correspond to three bond correlations, whereas in isomers **5** and **6**, the lactol proton and the hydroxymethine proton, respectively, were each four bonds distant from the acetal center. Furthermore, in planar structure **5**, an HMBC between the lactol proton and the alkene C-3 at 162.5 ppm would be anticipated.

**Chart 3:**
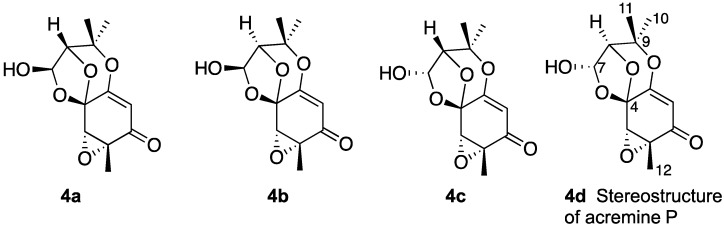
Candidate diastereomers of acremine P.

Therefore, planar structure **3** was considered further with DFT computations undertaken on four diastereomers (**4a**–**4d**) (Chart 3) following a conformational search on each of the four candidates. Diastereomers **4a** and **4d** had two contributing conformers, while **4b** and **4c** each had one contributing conformer. Three of the four candidates were reasonable matches to the ^13^C data, suggesting that the correct planar structure of acremine P had been elucidated; further, these data enabled diastereomer **4b**, for which the C-4 chemical shift was calculated at 116.5 ppm compared to an experimental value of 99.0 ppm, to be eliminated. It was not feasible to use the computational data to distinguish between the three remaining diastereomers owing to the similarity of their ^13^C-NMR shift values, and also to the errors inevitably inherent to the quantum mechanical calculations. It was noted that the quantum mechanical calculations revealed stereoisomers **4a** and **4d** to be the lowest in energy.

**Figure 1:**
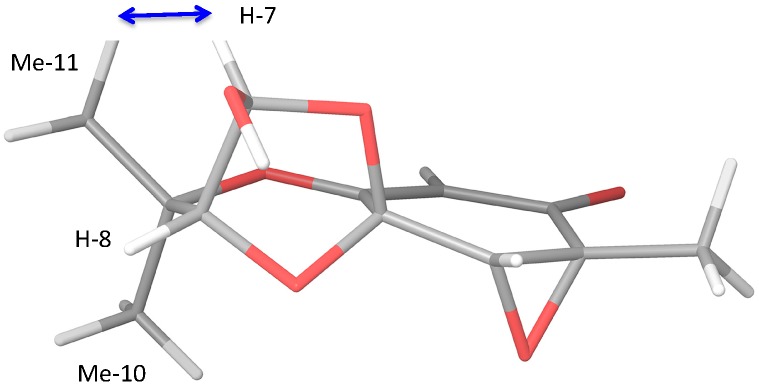
Three-dimensional image of stereoisomer **4d** showing nOe to Me-11.

The three remaining stereoisomers could be further distinguished by the zero coupling between the vicinal lactol and hydroxymethine protons, which necessitated a bond angle close to 90°. Selected homonuclear (H7–H8) couplings were calculated using the methods of Kutateladze et al. [15], yielding values of 0.2, 4.1, 3.9 and 0.3 Hz for *J*_H7–H8_ in stereoisomers **4a**–**4b**, respectively. The two stereoisomers (**4a**, **4d**) thus fitted the coupling data. It was noted that all four stereoisomers showed a heteronuclear (C4-H8) *J* value close to 5 Hz, and so these data did not distinguish **4a** from **4d**. NOe data also supported the choice of stereoisomer, in that from their 3D stereostructures both isomers **4a** and **4d** were expected to show an nOe between the lactol proton and one only of the two methyl groups; in contrast, isomers **4b** and **4c** were each expected to show nOes between the lactol proton and both methyl groups. In acremine P, the lactol proton at δ_H_ 5.83 shows an nOe to the methyl signal at δ_H_ 1.43 for H-11, but not to the methyl signal at δ_H_ 1.47 (Figure 1). The calculated chemical shifts were further examined using the DP4+ computational approach developed by Sarotti et al. to assign the most probable diastereomer [16]. Using the ^13^C-NMR data alone, the probability was 99.7% that **4d** was the correct diastereomer.

**Scheme 1:**
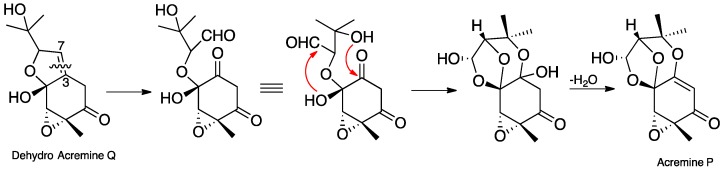
Putative biosynthetic pathway to acremine P (**4d**) from a dehydro derivative of acremine Q.

In our earlier publication [7], we reported that the hydrogenation of acremine P yielded acremine A (**2**) as the sole product, and used this information to specify the absolute configuration at C-6 of acremine P; clearly this information must be in error since the dioxolane ring of the revised structure is incompatible with the tetrahydrofuran ring previously ascribed to acremine P. Nevertheless, we anticipated that acremine P would have the same *6R* configuration as its co-metabolite acremine A. Scheme 1 shows a plausible biosynthetic route to the revised structure of acremine P. A didehydro derivative, generated by the action of a P450 enzyme on the co-metabolite acremine Q, could undergo oxidative ring cleavage of the C-3/C7 double bond [17], thereby generating an aldehyde group at the original C-7 position. The 1,3-dioxolane ring is formed by cyclization of the C-4 hydroxy group onto the aldehyde, and would be anticipated to provide the thermodynamically most stable lactol product. Finally, nucleophilic attack of the C-9 hydroxy group onto the C-3 carbonyl generates a hemiacetal intermediate; the subsequent dehydration step which generates an enol ether is facilitated by the associated formation of an α,β-unsaturated carbonyl.

## 3. Materials and Methods

The isolation and characterization of acremine P has been previously reported [7]. Details of computational calculations are given in the supplementary materials.

## 4. Conclusions

In conclusion, our revision of the structure of acremine P illustrates the valuable role of computational studies in evaluating the structures and stereochemistry of stereochemically complex natural products. At the same time, a single piece of data in the original study, i.e., the suggested conversion of acremine P into acremine A by hydrogenation, compromised the structural study and thereby incorrectly informed the structure determination.

## Figures and Tables

**Table 1:** Comparison of experimental vs. calculated ^13^C-NMR chemical shifts for acremine P (**4**). ^1^

Carbon	Exptl.	Calc. (1)	Calc. (4a)	Calc. (4b)	Calc. (4c)	Calc. (4d)
1	192.3, C	197.6	193.3	193.5	193.0	192.3
2	102.4, CH	122.8	108.7	104.0	108.7	102.4
3	162.5, C	159.0	164.6	163.3	165.5	162.5
4	99.0, C	108.0	101.3	116.5	101.0	99.0
5	59.1, CH	55.5	60.3	58.7	60.4	59.1
6	57.4, C	57.2	57.3	57.5	57.7	57.4
7	95.0, CH	72.0	95.9	99.4	98.8	95.0
8	86.2, CH	89.0	88.7	82.2	82.0	86.2
9	78.2, C	81.5	80.4	80.8	83.4	78.2
10	25.8, CH3	21.9	23.9	27.6	26.2	25.8
11	23.4, CH3	21.9	23.2	23.1	22.9	23.4
12	14.4, CH3	13.4	15.9	15.8	15.9	14.5

^1^ Carbon numbering selected so that carbon chemical shift values align with those provided in Reference [7].

## Supplementary Materials

Supplementary materials are available online.
