# Supplementary material for: Revision of the Structure of Acremine P from a Marine-Derived Strain of Acremonium persicinum
Source: Molecules. 2017 Mar 24;22(4):521. doi: 10.3390/molecules22040521 (PMC6154672; doi:10.3390/molecules22040521)
Supplement: Supplementary file 1 [file molecules-22-00521-s001.pdf]

Revision of the Structure of Acremine P from a Marine-Derived Strain of *Acremonium persicinum*

Mary J. Garson, [1,*] Warren Hehre,[2] Gregory K. Pierens, [3] and Suciati[4]

[1] School of Chemistry and Molecular Biosciences, The University of Queensland, Brisbane 4072 QLD, Australia; m.garson@uq.edu.au

[2] Wavefunction Inc., Irvine CA 92612, USA; hehre@wavefun.com

[3] Centre for Advanced Imaging, The University of Queensland, Brisbane 4072 QLD, Australia; greg.pierens@cai.uq.edu.au

[4] Faculty of Pharmacy, Airlangga University, Surabaya, East Java 60286, Indonesia; suciati@ff.unair.ac.id

* Correspondence: m.garson@uq.edu.au; Tel.: +61-3365-3605

Page 01 **Figure S1.** The image of the lowest energy optimized structure of **4d**.

Page 02 **Table S1.** Density functional theory calculations of coupling constants for **4a - 4d**

Page 03 **Table S2.** Computed coordinates of the lowest energy conformers of **4a** – **4d**

Figure 1. The image of the lowest energy optimized structure of **4d**.

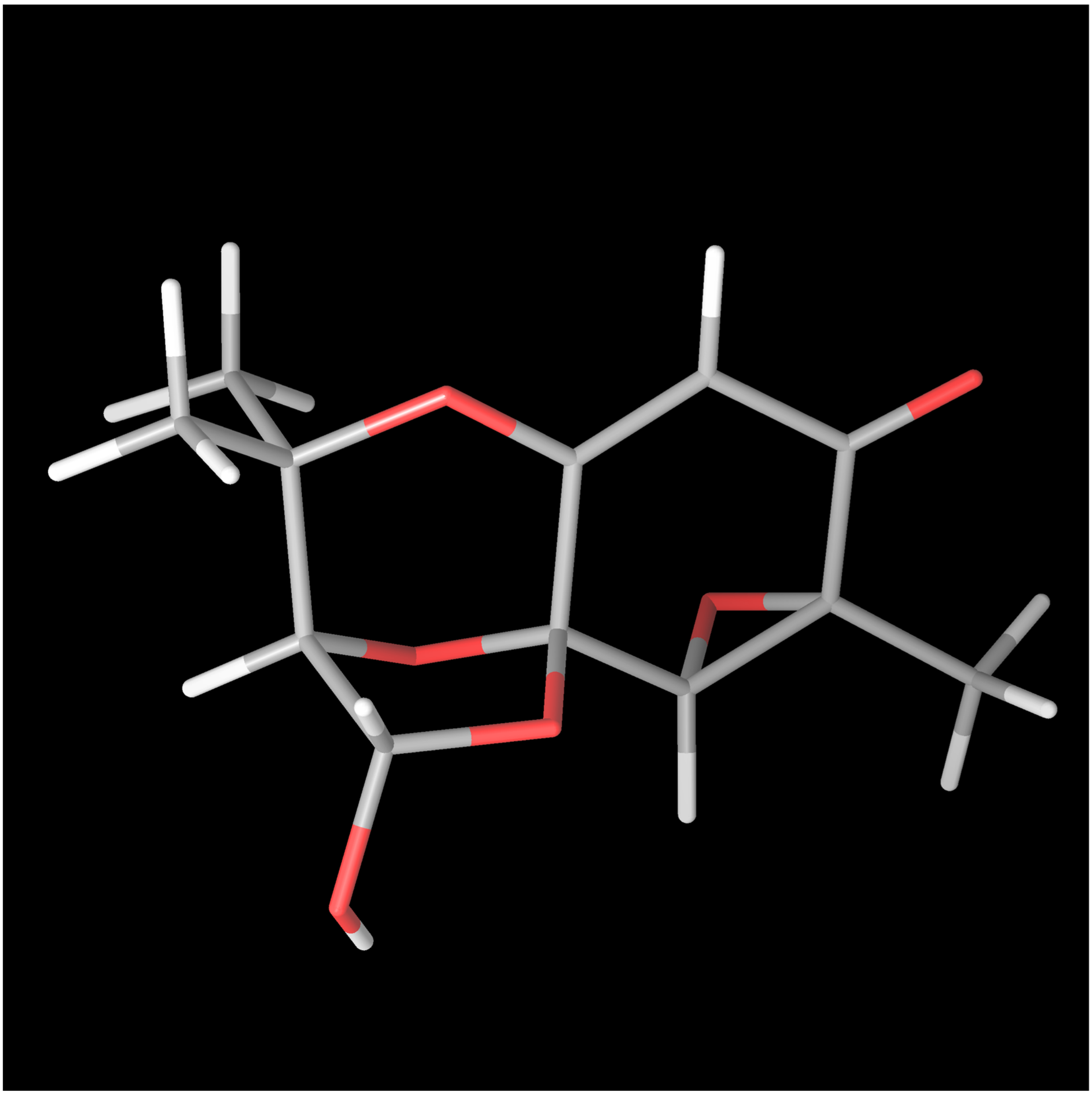

Table S1. Density functional theory calculations of coupling constants for **4a - 4d**

The ^{1}H-^{1}H and ^{1}H-^{13}C coupling constants were calculated using the DFT optimized structures as described by Kutateladze et al. (*J. Org. Chem.*, 2015, 80 (21), pp 10838–10848) using Gaussian 09W[#]. The data was extracted by the author supplied script and processed through the author's web site (http://kgroup.du.edu/nmr/).

Coupling Constant	Expt (Hz)	**4a** (Hz)	**4b** (Hz)	**4c** (Hz)	**4d** (Hz)
H7-H8	0	0.2	4.1	3.9	0.3
C4-H8	5.4	5.4	5	4.9	5.6

Gaussian 09, Revision D.01, M. J. Frisch, G. W. Trucks, H. B. Schlegel, G. E. Scuseria, M. A. Robb, J. R. Cheeseman, G. Scalmani, V. Barone, B. Mennucci, G. A. Petersson, H. Nakatsuji, M. Caricato, X. Li, H. P. Hratchian, A. F. Izmaylov, J. Bloino, G. Zheng, J. L. Sonnenberg, M. Hada, M. Ehara, K. Toyota, R. Fukuda, J. Hasegawa, M. Ishida, T. Nakajima, Y. Honda, O. Kitao, H. Nakai, T. Vreven, J. A. Montgomery, Jr., J. E. Peralta, F. Ogliaro, M. Bearpark, J. J. Heyd, E. Brothers, K. N. Kudin, V. N. Staroverov, T. Keith, R. Kobayashi, J. Normand, K. Raghavachari, A. Rendell, J. C. Burant, S. S. Iyengar, J. Tomasi, M. Cossi, N. Rega, J. M. Millam, M. Klene, J. E. Knox, J. B. Cross, V. Bakken, C. Adamo, J. Jaramillo, R. Gomperts, R. E. Stratmann, O. Yazyev, A. J. Austin, R. Cammi, C. Pomelli, J. W. Ochterski, R. L. Martin, K. Morokuma, V. G. Zakrzewski, G. A. Voth, P. Salvador, J. J. Dannenberg, S. Dapprich, A. D. Daniels, O. Farkas, J. B. Foresman, J. V. Ortiz, J. Cioslowski, and D. J. Fox, Gaussian, Inc., Wallingford CT, 2013.

Table S2. Computed coordinates of the lowest energy conformers of **4a** – **4d**

Compound **4a**

32
3a lower Energy

C	-0.24740	-0.76690	-1.97120
C	-0.47940	-0.83450	0.39420
O	0.77410	0.92400	-0.57720
C	-0.19280	0.65580	0.41700
C	-0.00700	0.73660	-1.76460
O	-0.91870	-1.29970	-0.79570
C	0.25430	1.18850	1.75130
C	0.47080	0.30130	2.90520
C	0.21840	-1.18720	2.72620
C	-0.33370	-1.64800	1.44760
C	1.08470	-1.49870	-2.12840
C	-1.17360	-1.05730	-3.14230
C	-1.26360	1.55070	-1.39930
O	-1.35610	1.35070	0.00060
O	0.51300	-1.96130	3.61730
O	-0.71720	1.09230	2.76830
C	1.39620	0.68050	4.02750
O	-1.15020	2.89710	-1.71320
H	-2.18220	1.20600	-1.87240
H	0.53250	1.16700	-2.60940
H	0.79170	2.13490	1.68910
H	-0.54120	-2.70840	1.35500
H	1.74440	-1.29490	-1.28170
H	0.90540	-2.57570	-2.18520
H	1.58990	-1.17950	-3.04570
H	-0.75700	-0.64640	-4.06720
H	-1.28030	-2.13870	-3.26260
H	-2.17010	-0.63800	-2.98700
H	2.39420	0.26740	3.85560
H	1.46510	1.76840	4.10640
H	1.02230	0.27380	4.96980
H	-0.38660	3.24030	-1.22810

32
3a higher Energy

C	-0.20670	-0.75970	-1.97590
C	-0.42840	-0.84400	0.39680
O	0.80140	0.91950	-0.57460
C	-0.15020	0.64810	0.42420
C	0.01980	0.74510	-1.75870
O	-0.86220	-1.30780	-0.79640
C	0.29960	1.18330	1.75540
C	0.51920	0.29400	2.90710
C	0.27040	-1.19570	2.72730

C	-0.27680	-1.65970	1.44780
C	1.13320	-1.47370	-2.14720
C	-1.13690	-1.05910	-3.14140
C	-1.24640	1.52350	-1.37950
O	-1.33180	1.32560	0.01730
O	0.56490	-1.96860	3.62000
O	-0.67140	1.08200	2.77470
C	1.44530	0.67350	4.02870
O	-1.04740	2.86140	-1.70400
H	-2.17100	1.13860	-1.82180
H	0.55040	1.19760	-2.59680
H	0.83130	2.13190	1.68960
H	-0.47360	-2.72200	1.35320
H	1.79650	-1.26440	-1.30480
H	0.96770	-2.55260	-2.20820
H	1.62670	-1.14250	-3.06660
H	-0.72940	-0.64160	-4.06750
H	-1.23180	-2.14150	-3.26330
H	-2.13720	-0.65130	-2.98050
H	2.44420	0.26360	3.85430
H	1.51170	1.76150	4.10850
H	1.07420	0.26440	4.97110
H	-1.75520	3.37070	-1.28890

Compound **4b**

32
3b

C	-1.65950	-0.37750	-1.68180
C	-0.54970	-0.82090	0.41290
O	-1.04170	1.43170	-0.25720
C	-0.09170	0.63060	0.40260
C	-0.91380	0.96700	-1.59870
O	-1.06760	-1.29970	-0.73060
C	0.20510	1.19400	1.76350
C	0.43540	0.30620	2.91080
C	0.28600	-1.19270	2.69520
C	-0.34760	-1.64120	1.45640
C	-1.59120	-1.06140	-3.04020
C	-3.11910	-0.16750	-1.26790
C	0.63050	0.94610	-1.73360
O	1.07390	0.73270	-0.40010
O	0.66870	-1.96470	3.55570
O	-0.79920	1.02250	2.73690
C	1.29590	0.72770	4.06840
O	1.08250	-0.03590	-2.59700
H	0.99840	1.93440	-2.03940
H	0.68360	2.17210	1.72870
H	-0.61740	-2.68920	1.38840
H	-0.57010	-1.33650	-3.29450
H	-2.21380	-1.96050	-3.02020
H	-1.98400	-0.39170	-3.81240
H	-3.63390	0.47190	-1.99300
H	-3.18700	0.29710	-0.28240
H	-3.62530	-1.13610	-1.24030
H	1.28040	1.81560	4.17160
H	2.32600	0.39410	3.91530
H	0.92980	0.27190	4.99110
H	2.04740	-0.02230	-2.58160
H	-1.37710	1.69800	-2.26360

Compound **4c**

32
3c

C	-0.23270	-0.75490	-1.97880
C	-0.47030	-0.83760	0.38820
O	0.72840	0.95810	-0.59280
C	-0.20820	0.65880	0.42120
C	-0.06470	0.76310	-1.76780
O	-0.87830	-1.31760	-0.80310
C	0.26230	1.18670	1.75000
C	0.47650	0.30080	2.90410
C	0.20670	-1.18550	2.73050
C	-0.32940	-1.64720	1.44750
C	1.14920	-1.40200	-2.10830
C	-1.10380	-1.13640	-3.16650
C	-1.32370	1.55520	-1.35800
O	-1.38350	1.35530	0.04560
O	0.48110	-1.95680	3.63140
O	-0.70390	1.10460	2.77560
C	1.41440	0.67230	4.01860
O	-2.47420	1.12410	-1.99270
H	-1.16410	2.62870	-1.53160
H	0.46030	1.21130	-2.61310
H	0.80660	2.12800	1.67860
H	-0.52330	-2.70990	1.35110
H	1.03290	-2.48770	-2.16250
H	1.64490	-1.05950	-3.02250
H	1.78670	-1.15700	-1.25610
H	-1.09300	-2.22410	-3.28250
H	-0.70080	-0.69120	-4.08210
H	-2.12820	-0.79850	-3.02880
H	2.40740	0.25020	3.83890
H	1.49390	1.75960	4.09580
H	1.04420	0.26920	4.96380
H	-3.21830	1.60630	-1.61090

Compound **4d**

32
3d lower Energy

C	-1.64750	-0.39420	-1.68360
C	-0.53390	-0.82290	0.41070
O	-1.11640	1.41770	-0.20320
C	-0.11340	0.63640	0.40480
C	-1.00390	0.99290	-1.56090
O	-0.99390	-1.30450	-0.76070
C	0.21540	1.19370	1.76080
C	0.44320	0.30540	2.90870
C	0.27140	-1.19240	2.70260
C	-0.34520	-1.64420	1.45370
C	-1.46580	-1.00750	-3.06410
C	-3.12410	-0.32340	-1.29770
C	0.52860	1.05130	-1.75220
O	1.01350	0.75570	-0.44850
O	0.62920	-1.96490	3.57220
O	-0.78320	1.03590	2.74270
C	1.31800	0.71720	4.05880
O	0.97540	2.29100	-2.18070
H	0.92710	0.32370	-2.45860
H	-1.51630	1.71310	-2.20100
H	0.70710	2.16540	1.72330
H	-0.59750	-2.69590	1.37670
H	-1.96330	-1.98040	-3.10050
H	-1.91260	-0.36240	-3.82710
H	-0.41130	-1.16080	-3.30530
H	-3.54970	-1.33040	-1.29930
H	-3.67860	0.29140	-2.01440
H	-3.24240	0.11010	-0.30200
H	1.32600	1.80580	4.15510
H	0.94690	0.27480	4.98580
H	2.34030	0.36180	3.90260
H	0.68120	2.94260	-1.52840

32
3d higher Energy

C	-0.87110	0.11730	-2.00640
C	-0.83320	-0.40970	0.35100
O	0.68880	1.19560	-0.55210
C	0.57800	0.15400	0.37980
C	0.60180	0.48470	-1.78240
O	-1.33730	-0.66520	-0.87310
C	1.03610	0.61890	1.73220
C	0.31760	0.21430	2.94870
C	-0.93960	-0.62950	2.79430
C	-1.50780	-0.77120	1.45210
C	-1.08880	-0.76080	-3.22960

C	-1.72310	1.38330	-2.07800
C	1.53760	-0.70510	-1.50410
O	1.44400	-0.86850	-0.09780
O	-1.44390	-1.14370	3.77550
O	0.17990	1.52860	2.38540
C	1.01250	0.12510	4.27820
O	2.82100	-0.34750	-1.89630
H	1.22580	-1.64760	-1.96660
H	0.98160	1.11840	-2.58450
H	2.10180	0.84000	1.77560
H	-2.48280	-1.23890	1.37200
H	-2.15550	-0.97270	-3.34260
H	-0.74170	-0.24530	-4.13060
H	-0.56470	-1.71570	-3.14590
H	-2.77930	1.10860	-2.14190
H	-1.46050	1.97190	-2.96330
H	-1.56950	2.00180	-1.19100
H	1.86320	0.81070	4.30460
H	0.31690	0.38070	5.08060
H	1.36600	-0.89490	4.45280
H	3.42640	-1.03750	-1.59650
